# The BRI-on-chip disposable test device opens up substantial savings potentials in urolithiasis treatment

**DOI:** 10.1186/1878-5085-5-S1-A56

**Published:** 2014-02-11

**Authors:** Norbert Laube, Wolfgang Berg, Thomas Knoll, Heinz Busch

**Affiliations:** 1 NTTF Coatings GmbH, D-53619 Rheinbreitbach, Germany; 2 Klinik und Poliklinik für Urologie, Universitätsklinikum Jena, D-07743 Jena, Germany; 3 Klinikum Sindelfingen-Böblingen, Sindelfingen, D-71065 Sindelfingen, Germany

## Scientific objectives

Urolithiasis is a widespread disease in the developed countries with rising prevalence rates which presently lie at least at 5%. Thus, 25 Mio. EU-citizens suffer from that symptom which burdens the healthcare systems by several tens of billions of Euros yearly. Stone formation is the result of an altered urinary composition, mostly caused by an underlying metabolic disease and disadvantageously interacting external risk factors. This makes the pathogenesis in each patient unique and thus diagnosis, causal treatment and therapeutic monitoring difficult, costly and time-consuming. Mostly, no specific long-term therapy for prevention of recurrence follows the purely symptom-based stone removal.

**Figure 1:**
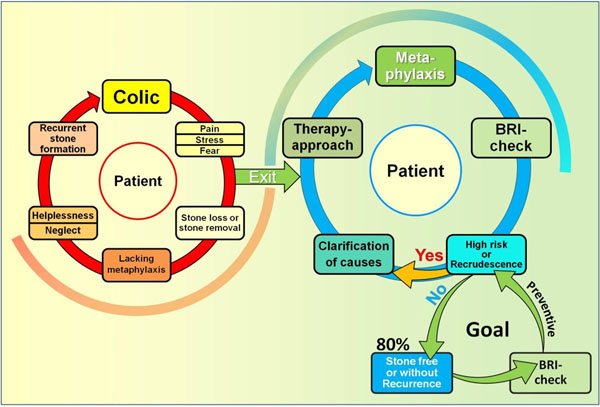
The BRI facilitates to leave the vicious circle.

The consequence is a diagnostic and therapeutic under-supply which inevitably results in a vicious circle of recurrent stone formation, although the recurrence rate can be lowered from 80% without metaphylaxis down to 20% under risk-adopted therapy. A paradigm shift from “stone-removal-only” towards a predictive, preventive and personalized treatment according to guideline recommendations is required. Routine urinalysis is restricted to only a few selected lithogenically important constituents whose concentrations partly can be combined to indices. However, to date no simple, cost-effective and precise method to determine the crystal formation risk of a native urine exists on the market.

## Technological approaches

The scientifically established Bonn-Risk-Index (BRI) is based on a crystallization test induced within a sample of native urine. As urine composition reflects the influence of any of the patient’s metabolic processes – even of those analytically not directly accessible – BRI reflects a patient’s risk situation on a highly personalized level. BRI has proven its excellent appropriateness by showing diagnostic sensitivity and specificity clearly exceeding even those of the most evolved analysis-based risk indices. Whereas BRI can be cheaply and beneficially determined within a few minutes, standard urinalysis needs unacceptable amounts of time and costs. In 2008 the “Urolizer” mini-lab for fully-automated BRI measurement was developed; some devices are still in use. Experience shows that the requirements of medical laboratories, hospitals and medical offices differ in part. Whereas the former are very satisfied about the Urolizer, the latter two asked again for a simplified BRI test device.

## Results interpretation

In preliminary tests the basic feasibility of an alternative approach of BRI-determination was demonstrated. Currently, a miniaturized point-of-care test device is being developed. The disposable “BRI-on-Chip” meets particularly the requirements of the resident doctors. The result is presented in three levels within a few seconds.

## Outlook

The BRI-on-Chip approach perfectly fits both the medical and socio-economical needs as: (1) an inexpensive, reliable, intuitively and easy-to-use diagnostic tool for the evaluation of the urinary stone formation risk is given to the physicians allowing them to better apply an appropriate stone metaphylaxis, and (2) it helps to reduce the continuously increasing urolithiasis-related costs as the recurrence rates will be consecutively lowered. It can be expected, that the BRI-principle allows also for the screening of other metabolic diseases.

